# Comparability of tympanic and oral mercury thermometers at high ambient temperatures

**DOI:** 10.1186/1756-0500-5-356

**Published:** 2012-07-16

**Authors:** Amy L Chue, Rachael L Moore, Andrew Cavey, Elizabeth A Ashley, Kasia Stepniewska, François Nosten, Rose McGready

**Affiliations:** 1 Shoklo Malaria Research Unit, 68/30 Baantung Road, PO Box 46, Mae Sot, Tak 63110, Thailand; 2 Mahidol-Oxford Clinical Research Unit, Faculty of Tropical Medicine, Mahidol University, 420/6 Ratchawithi Road, Ratchathewi, Bangkok 10400, Thailand; 3 Centre for Clinical Vaccinology and Tropical Medicine, Churchill Hospital, Oxford OX3 7LJ, UK

**Keywords:** Ambient temperature, Oral mercury thermometer, Tympanic thermometer, Tropical setting

## Abstract

**Background:**

Body temperature can be measured in seconds with tympanic thermometers as opposed to minutes with mercury ones. The aim of this study was to compare tympanic and oral mercury thermometer measurements under high ambient field temperatures.

**Results:**

Tympanic temperature (measured thrice by 3 operators) was compared to oral temperature measured once with a mercury-in-glass thermometer in 201 patients (aged ≥5 years), on the Thai-Myanmar border. Ambient temperature was measured with an electronic thermo-hygrometer. Participants had a mean [min-max] age of 27 [5–60] years and 42% (84) were febrile by oral thermometer. The mean difference in the mercury and tympanic temperature measurement for all observers/devices was 0.09 (95%CI 0.07-0.12)°C and intra-class correlation for repeat tympanic measurements was high (≥0.97) for each observer. Deviations in tympanic temperatures were not related to ambient temperature.

**Conclusion:**

Clinically significant differences were not observed between oral and tympanic temperature measurements at high ambient temperatures in a rural tropical setting.

## Background

The easy-to-use infrared tympanic thermometer requires only seconds to take a body temperature measurement [1,2]. This device boasts reliable and accurate temperature readings, within ±0.2°C [3], the usual accepted range in clinical practice [4]. Infrared thermometers measure the radiant heat emitted from the tympanic membrane [5]. The tympanic membrane and hypothalamus share an arterial supply from branches of the carotid artery hence the tympanic membrane is a direct reflection of the core temperature [6-8]. The value of tympanic thermometers in clinical practice is inconsistent due to variations between right and left ears and poor repeatability [9]. However, there is evidence to suggest that training in measuring tympanic temperature increases repeatability and that hand dominance may have an effect on right and left ear variability [9]. Body temperature is thought to be fluctuant and is dependent on age, gender and site of measurement, with wide changes due to inter- and intra-individual variability [9]. Sund-Levander et al. therefore suggest that “…body temperature should be evaluated in relation to individual variability, a baseline value, and the best approach is to measure it at the same site without adjustments to other sites…” [9].

Simulations of extremes of temperature have been attempted, with one study finding a significant increase in both oral and tympanic temperatures, which persisted for 20 minutes (with initial tympanic elevation being greater than oral), after exposure to an environment controlled at 43.5°C [6]. No studies have assessed the suitability of infrared tympanic thermometers in the rural tropics where high ambient temperatures are pervasive. The aim of this study was to compare the temperature obtained by use of tympanic thermometers to oral mercury thermometers, still widely used in resource poor settings.

## Methods

### Ethics

The study was approved by the local Thai-Myanmar border Community Advisory Board. After a brief verbal description willingness to participate was also confirmed verbally.

### Population

Participants of Karen and Myanmar ethnicity were enrolled in the Shoklo Malaria Research Unit clinic in Wang Pha, 20 km north of the Thai town of Mae Sot, on the Thai-Myanmar border in April 2005.

### Inclusion and exclusion criteria

The age of 5 was chosen as a cut-off, as children under this age are unable to hold an oral thermometer properly in their mouths in this setting. Severely unwell patients or those with visible ear discharge were excluded.

### Sample size calculation

A sample size of 203 would be sufficient to detect a 0.2°C difference between the method of temperature measurement, on the same patient, using 90% power and a 5% significant level (standard deviation 0.88°C).

### Data collection

Three members of staff were involved in measuring temperatures. They participated in a workshop on correct technique and had at least one month’s experience with the tympanic thermometer. For oral temperature measurements, a new *Dura* mercury-in-glass thermometer (manufactured by Safety Co, LTD) was used. The thermometer was placed sublingually by a single observer, timed for five minutes, handed to a different staff member, who read and cleaned the thermometer, and recorded ambient temperature from an electronic thermo-hygrometer, (Model JB 913R, manufactured by Irox; manufacturer reported error ±1°C, from 0–50°C). Five minute readings were chosen as previous literature reported stabilisation of temperature often takes over three minutes [10,11].

During the five minute period, tympanic temperatures were measured directly using an infrared ear thermometer (*Braun ThermoScan* IRT 4520), with a manufacturer reported accuracy of ±0.2°C, in the range of 34–42.2°C, and ±0.3°C outside this range [3]. Each observer was allocated a new tympanic thermometer for the survey. Temperatures were taken in accordance with the manufacturer’s directions and thermometers calibrated in degrees Celsius. As tympanic temperatures were measured directly, adjustment to other sites was not required. The right ear was chosen as this was the side for routine physical examination. The ear tug technique [12] was applied. Observers were blind to the readings of other observers. The first observer recorded on their own sheet of paper the first three temperature measurements taken on the right ear during a 20 second interval. This was then repeated by the second and third observers in the same order each time, to a total of nine measurements per participant. Calibration of the three “off-the-shelf” thermometers was assumed to be accurate.

### Statistical analysis

Results were analyzed using SPSS, version 18.0 for Windows, and STATA software. The agreement between the oral and tympanic temperature readings, and between observers using three different devices, was analyzed by the technique described originally by Bland and Altman [13]. Agreement between tympanic and oral measurement methods was accepted when the mean ±2 standard deviations was within the clinically accepted ±0.2°C [4]. A random effects model, with nested random effects attributable to observer (tympanic thermometer) and occasion (first, second or third measurement), was fitted to model a difference between the tympanic and oral temperatures, adjusted for the ambient temperature.

## Results

### Demographic information

In April 2005 during the two weeks of the study, 72.6% (204/281) of patients aged >5 years consented to participate. There were 201 oral and 1809 tympanic temperature measurements analysed as 1.3% (3/204) did not keep the oral thermometer in their mouths for five minutes. The mean age (±standard deviation [min-max]) was 27 ± 12 [5–60] years, of whom 40.8% (82/201) were male, 26.9% (54/201) healthy pregnant women, and 38.3% (77/201) were febrile (defined as a temperature of >37.5°C) by the oral thermometer measurement.

### Agreement between oral and tympanic temperature measurement

**Figure 1:**
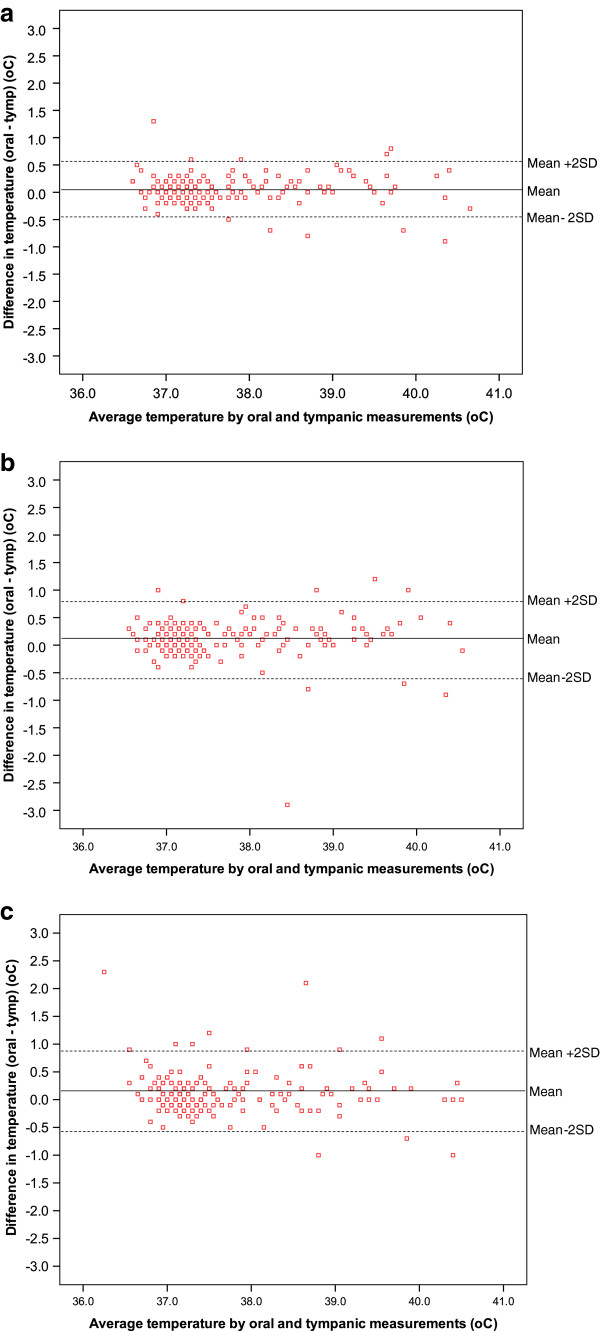
a-c. Agreement between oral and first tympanic temperatures for observer 1(a), observer 2(b) and observer 3(c).

The mean difference (95%CI) in the oral and first tympanic temperature measurement for each observer (1, 2 and 3) was: 0.05 (0.01-0.08)°C, 0.11 (0.07-0.16)°C, 0.12 (0.07-0.17)°C, respectively (Figures 1a-c). The mean difference for observer 1 was significantly lower than the mean for observer 2 and 3 (*P* = 0.025, *P* = 0.022) but not for observer 2 and 3 (*P* = 0.879).

### Intra-observer/device variation and intra-class correlation coefficient

The mean difference between tympanic measurements for each observer/device was small (Table 1). Intra-class correlation coefficient (95%CI) of the three measures taken by each observer/device was high (Table 1). Intra-class correlation coefficient (95%CI) between the average of the three measures made by each observer/device was also high: 0.956 (0.944-0.965).

**Table 1:** The mean difference (95%CI) between the three tympanic measurements made by each of the 3 observers/devices and the intra-class correlation coefficient (ICC)

	Mean difference ± standard deviation (95%CI)^*			**ICC (95%CI)**
**Observer/device**	**Reading 2 vs 1**	**Reading 3 vs 2**	**Reading 3 vs 1**	
	**N = 201**	**N = 201**	**N = 201**	
1	0.03 ± 0.01	0 ± 0.09	0.04 ± 0.12	0.993
	(0.02 to 0.05)	(0 to 0.02)	(0.02 to 0.05)	(0.991-0.994)
2	0.03 ± 0.12	0.01 ± 0.12	0.03 ± 0.15	0.979
	(0.01 to 0.05)	(−0.01 to 0.02)	(0.01 to 0.05)	(0.962-0.977)
3	0.05 ± 0.22	−0.02 ± 0.16	0.03 ± 0.26	0.971
	(0.02 to 0.09)	(−0.04 to 0)	(0 to 0.07)	(0.964 -0.977)

^ Temperature in degrees Celsius.

* The mean difference is calculated by subtracting the later reading from the former reading, in the order they were taken e.g. Reading 2 vs 1, is the mean difference of the second temperature reading minus the first temperature reading.

Most (92.0% (1,665/1,809)) differences between tympanic temperature measurements on the same subject were within the manufacturers reported accuracy of ±0.2°C, and 98.4% (1780/1809) with ±0.5°C.

### Ambient temperature

**Figure 2:**
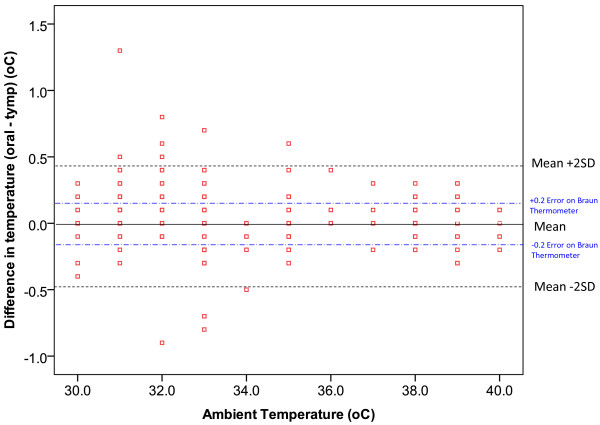
Difference between oral and tympanic temperatures measured at varying ambient temperatures.

The correlation between tympanic temperature measurement taken by each observer and the ambient temperatures was low, indicating the tympanic thermometer is reliable at high ambient temperatures (≥30°C) (Figure 2). In the random effect model we found that on average the difference between oral and tympanic temperatures was 0.07 (0.02-0.11)°C with a standard deviation of 0.30°C. The variability between observers and occasions was negligible: the standard deviation was 0.04°C and 0.002°C respectively. Ambient temperature affected the difference only slightly (*P* = 0.002) with the difference between the two methods of temperature measurement being 0.09 (0.04-0.15)°C at 30°C ambient temperature and 0.04 (−0.01-0.09)°C at an ambient temperature of 40°C.

## Discussion

No clinically relevant significant difference was observed between oral and tympanic temperature measurements at high ambient temperatures. This agrees with previously published reports from temperate climates [14-17] and studies that simulated high ambient temperatures [6]. The random effects model reassuringly demonstrated that high ambient temperature had a minimal effect on tympanic measurements in this setting. A high intra-class correlation coefficient (≥0.97) suggests that almost all of the variation observed was due to differences between patients rather than differences in the repeated measurements taken by each observer on any one patient. It also suggests that new tympanic thermometers are reliable as stated in the product information leaflet. A small proportion (1.6%) of inter-observer measurements varied by >0.5°C, possibly due to poor probe positioning, handedness or ear wax and data recording errors cannot be ruled out [7,12,16,18-21]. Measuring right ear temperature with the right hand and taking the average of two measurements can minimize such errors [7,19]. Informal assessment found health workers adapted to the use of tympanic thermometers quickly with the main problem in ongoing quality control being a failure to conduct the ear tug in older patients. However, the cost of a tympanic thermometer is high and for this reason, they are not often used in resource poor settings. In Thailand, the current 25 baht cost of the oral mercury thermometer is much lower than the 2,152 baht for the tympanic thermometer, but the price of health worker time consumed, 5 minutes versus 5 seconds, was not quantified.

Mercury-in-glass thermometers have been used to measure temperature since 1867 [4], however, the hazards of mercury leakage, cross infection, and the time required for measuring and reading, makes it all the less appealing, resulting in the development of digital and infrared thermometers over recent years [4,22]. The use of mercury thermometers has also been discontinued by several countries in Europe and some states in the United States due to the risk of mercury poisoning [23]. Children below the age of 5 years were not included and they form a large part of outpatient services in the rural tropics. Each patient was not checked for ear wax (may cause a possible difference in temperature of 0.13-0.3°C [18]); and calibration of the thermometers was assumed. Only a single measurement of the mercury thermometer was done precluding analysis of intra- and inter-observer variability.

## Conclusion

The tympanic thermometer provides comparable but more rapid results than the oral mercury thermometer even with high ambient temperatures in the rural tropics.

## Competing interests

The authors do not have competing interests.

## Authors’ contributions

ALC analysed and interpreted the data and drafted and revised the manuscript. RLM contributed to the conception and design and the acquisition of data. AC contributed to the conception and design and the acquisition of data. EAA contributed to the conception and design and the acquisition of data and drafted the manuscript. KS analysed and interpreted the data and drafted the manuscript. RM contributed to the conception and design and the acquisition of data, analysed and interpreted the data and drafted and revised the manuscript. FN revised the manuscript. All authors read and approved the final manuscript.
